# Evaluation of the Impact of Irradiance Lamps and Storage Media on Elution of TEGDMA from Dental Composites

**DOI:** 10.1055/s-0044-1786876

**Published:** 2024-05-24

**Authors:** Kiran Tauseef, Faiza Amin, Syed Faraz Moin, Zohaib Khurshid, Kashif Aslam, Bushra Jabeen

**Affiliations:** 1 Department of Dental Material, Dow Dental College, Dow University of Health Sciences, Karachi, Pakistan; 2 Dr. Zafar H Zaidi Centre for Proteomics, University of Karachi, Karachi, Pakistan; 3 Department of Prosthodontics and Dental Implantology, College of Dentistry, King Faisal University, Al Ahsa, Saudi Arabia; 4 Department of Prosthodontics, Dow Dental College, Dow University of Health Sciences, Karachi, Pakistan; 5 Department of Prosthodontics, Dow International Dental College, Dow University of Health Sciences, Karachi, Pakistan

**Keywords:** degree of conversion, light curing units, dental composites, elution of monomer, high-performance liquid chromatography

## Abstract

**Objectives** The aim of this study was to evaluate and compare the effect of irradiance light and storage media on the elution of triethylene glycol dimethacrylate (TEGDMA) from conventional Filtek Z350XT 3M ESPE and two bulk-fill composites Shofu Beautifil-Bulk and Filtek Bulk fill flowable 3M ESPE using high-performance liquid chromatography (HPLC).

**Materials and Methods** Shofu Beautifil-Bulk, Filtek Bulk fill flowable 3M ESPE, and Filtek Z350XT 3M ESPE were the three types of composites used in this study. Disk shaped samples of 4-mm thickness and 10-mm diameter were fabricated using a stainless steel mold and were polymerized using light emitting diode (LED) and quartz tungsten halogen (QTH) lamps. After polymerization, the samples were immersed in ethanol, artificial saliva with betel quid extract, and distilled water for 1, 7, and 30 days, respectively. The elution of monomer TEGDMA was evaluated using HPLC.

**Statistical Analysis** To evaluate the mean concentration difference, mixed way analysis of variance (ANOVA) was applied. Between different light, materials, and within the time duration, Tukey's post hoc test was used. A *p* value of 0.05 was considered significant.

**Results** During the first day of storage, a significant amount of monomer TEGDMA elution was seen in all the materials. The highest values observed to be in the disks cured with QTH lamp. However, the highest elution was seen when the disks were immersed in ethanol/water solution. While the most stable medium was distilled water, artificial saliva with betel nut extract also had a significant effect on the elution of TEGDMA. The highest value obtained was of Filtek Bulk fill flowable 3M ESPE after 30 days of immersion in both LED and QTH cured disks.

**Conclusion** Filtek Bulk fill flowable 3M ESPE shows better properties in relation to the release of monomer TEGDMA as it releases less amount of monomer in the storage media. The release of monomer was highest in ethanol as compared to artificial saliva and distilled water with the passage of time.

## Introduction

Dental composites are composed of three major components that are chemically different from each other. These materials are used for restoration of the loss of tooth structure due to caries, trauma, or other diseases. These materials include resin matrix of natural polymerizable structure, inorganic filler for strengthening, and a coupling agent known as silane coupling agent. The coupling agent enhances the chemical bonds between the organic matrix and inorganic fillers. 1 Base monomers such as bisphenol A glycidyl methacrylate (Bis-GMA) and urethane dimethacrylate (UDMA) along with low-viscosity monomer like triethylene glycol dimethacrylate (TEGDMA) constitute the polymerizable resin matrix. Composites can also be used to elute and to cement crowns and veneers. They are also indicated for the periodontal splinting, noncarious lesions, enamel hypoplasia, and repair of old composites. 1 Dental composites are considered among the most adaptable dental filling materials. It is being extensively used in dentistry since its introduction in the dental market about 50 years ago. 2

The common drawbacks of composites include polymerization shrinkage and high residual monomer content. 3 The elution of residual monomers, oligomers, and other degradation products into the oral environment has raised concerns regarding composites. It occurs by diffusion of the resin matrix or by its degradation or erosion over the period. 3 In addition to their effect on mechanical properties such as decreased wear resistance, hardness, and increased tendency to discoloration, eluted monomers can contribute to a variety of local or systemic health effects. 4 5 They can be released either into the oral cavity or diffuse into the pulp through dentinal tubules causing local reactions including pulpal irritation, allergenic, cytotoxic, and genotoxic effects. Eluted TEGDMA has been shown to promote the growth of cariogenic bacteria. Some eluted substances have been linked to systemic effects. Bisphenol A (BPA), a hydrolytic degradation product or a contaminant eluted from aromatic-based systems, has been confirmed to have parahormonal activity and it can imitate hormones from the estrogen group and thus may contribute to female infertility. 6

Dentists use incremental placement techniques to reduce polymerization shrinkage. This technique is time-consuming, and there is risk of contamination while placing, adapting, and curing each increment. Recently, a new class of resin-based composite, called “bulk-fill” composites, has been introduced with the purpose of saving clinical chair side time. Its unique advantage is that it can be placed and cured in an increment of 4-mm thickness, without undergoing polymerization shrinkage. 7

The degree of conversion (DC) of a resin composite is crucial in determining its biocompatibility. 8 It has been shown that decrease in DC might lead to a decrease in the physical/mechanical properties and an increase in the elution of monomers, as well as negatively affecting the pulp tissue. It has been found in various studies that a significant amount of organic compound residue remains unbound in the cured material. 9

As described by Ferracane, 2 several factors can influence the elution of different compounds from resin-based dental materials. First, the amount of compounds released is directly related to the DC, which varies between 50 and 70% and reaches a maximum after 24 hours due to a postcure process. 10 11 Second, the type of extraction solution can affect the elution. Third, the size and chemical nature of the released components play a role. Additionally, the physical and mechanical characteristics of the composite resins are related to the filler content, filler size, and distribution of filler particles. Therefore, the composition (filler content) of composites can directly influence the elution process. 12

According to Sajnani and Hegde, 3 the DC for dental composites is approximately between 35 and 77%. The storage solutions and chemistry of the solvent have got a direct impact on the elution of unreacted monomer. Łagocka et al 13 reported that the type of extraction medium used impacts both the concentration of the eluted monomer and the elution time. Generally, the degree of monomer elution is proportional to the hydrophobicity and swelling capacity of the organic solvent used.

The qualitative and quantitative methods of analyzing unreacted monomers and degradation products include Gas Chromatography (GC) high-performance liquid chromatography (HPLC), GC/mass spectrometry, and electrospray ionization/mass spectrometry. HPLC is the technique used most often. 8 Both HPLC and GC can be coupled to a mass spectrometer (MS) as a detector to increase the sensitivity and selectivity of the technique. This also allows identification of unknown substances and degradation products from both uncured resins and extracts from cured materials. 14

Despite multiple benefits of resin-based composites, there are few drawbacks associated with it. This monomer possesses different types of toxic effects; particularly, they produced genotoxic and cytotoxic effects. As a result of this toxicity, pulp formation and multiple allergic reactions along with infections have been observed within the patient's physical, chemical, and mechanical properties of the filling material. 2

Therefore, the aim of this study was to evaluate different composites and how different factors including storage time, storage solution, and curing modes affect the monomer release. Consequently, the study focused on evaluating the release of TEGDMA under different conditions using HPLC.

## Material and Method

### Resin-Based Composite's Composition and Preparation

In total, 180 disk-shaped samples were prepared using a stainless steel mold of 4-mm thickness and 10-mm diameter (ISO 4049). Conventional nanohybrid composite (Filtek Z350XT 3M ESPE) was used in the plays control group and two bulk-fill composites (Shofu Beautifil-Bulk and Filtek Bulk fill flowable 3M ESPE) were used by the experimental groups in the study. The composite materials, their composition, batch numbers, and manufacturers are given in Table 1 .

**Table 1:** Composites used in the study

Material	Type	Composition	Processing method	Manufacturer
Composite (Filtek Z350XT), control group	Conventional nanohybrid composite	Resin matrix contains Bis-GMA, DMA, TEGDMA, and PEGDMA Fillers: agglomerated/nonaggregated 20-nm silica filler, nonagglomerated/nonaggregated 4- to 11-nm zirconia filler, and aggregated zirconia/silica cluster filler	Placed in increments of 2 mm and cured 9	3M ESPE Batch no: N162941
Filtek Bulk fill flowable (group B2)	Bulk fill flowable composite	Matrix: Bis-GMA, UDMA, Bis-EMA, and TEGDMA Zirconia filler, ytterbium trifluoride (YbF3) has been added to increase the radiopacity. The inorganic filler loading is ∼64.5% by weight (42.5% by volume)	Placed in bulks of 4 mm 9	3M ESPE Batch no: 1506500564
Beautifil-Bulk (group B1)	Bulk fill composite	Resin-based matrix: Bis-GMA; UDMA; Bis-MPEPP; TEGDMA Filler: flouroboroaluminosilicate glass	Placed in bulks of 4 mm 9	Shofu Batch no: PN 2035

Light emitting diode (LED) light (Mectron S.p.A carasco GE, Italy) with a wavelength between 440 and 480 nm with the narrowest peak at 460 nm and an output irradiance greater than 1,400 mW/cm ^2^ was used. A quartz tungsten halogen (QTH) lamp (DeepBlue Technology Co., Ltd) with an illumination intensity of 480 mW/cm ^2^ and output voltage of 12 V and output power 75 W was used to cure the samples for 40 seconds from the topmost surface.

The mold was positioned over a cellulose acetate strip on a glass slab. The cellulose acetate strip resting on top of the composite prevents the formation of an oxygen inhibition layer. A matrix strip and a glass slab with firm pressure were placed for removal of excess material. Excess material was removed using a blade to prevent overfill. Each sample was softly pressed out from the mold after curing and flash of the material was detached by means of a blade. The disks were then finished and polished to attain a smooth surface without any voids or irregularities.

After curing of the samples ( *n* = 10), samples from each material were immersed into dissimilar media enclosed in closed vials. Each sample was stored in 2 mL of storage medium at 37°C in an incubator. The solutions used in the study are detailed in Table 2 .

**Table 2:** Solutions used in the study

1	TEGDMA (triethylene glycol dimethacrylate) standard monomer: 250 mL; Sigma Aldrich, Germany
2	Ethanol (BDH-analytical grade)
3	Artificial saliva (MERCK) with betel quid extract 250 mL (AKUH Pharmacy); 5 mg of split beetle nut was added in the bottle of artificial saliva; pH of artificial saliva was 6.8
4	Distilled water (MERCK Millipore Ultrapure type 1 water)
5	Acetonitrile (MERCK)

The ratio between the sample and the medium volume was greater than 1:10. The disks were entirely immersed in the medium, in line with the requirements of ISO 10993-12. 6

Sorbitol was not used in artificial saliva as it would result in a more glutinous solution than natural saliva. 6 Storage samples were collected after 1 day and replaced with a fresh solution. After 7 days of storage, samples were collected again for testing and analyzed for the elution of unreacted TEGDMA. The storage medium was then refreshed again and analyzed after 30 days for elution of TEGDMA. The same protocol was followed for sample fabrication of both experimental groups.

All the collected solutions after a specific period of storage were transferred into HPLC vials and assessed by PerkinElmer USA, series 200 for elution of TEGDMA. The detector used was series 200 UV/VIS. The wavelength of 205 nm was used. Samples (10 µL) were loaded using a series 200 auto-sampler and the software used for data acquisition was TotalChrom. The separation of monomer was carried out using SPHERl-5ODS C18 column (5 µm) 250 × 4.6 mm using a solvent system of 80% acetonitrile (analytical grade, Merck) and 20% deionized water (Millipore) as solvents. The mobile phase was run at a flow rate of 1 mL/min. Isocratic program was run for 10 minutes for all the samples.

**Fig. 1:**
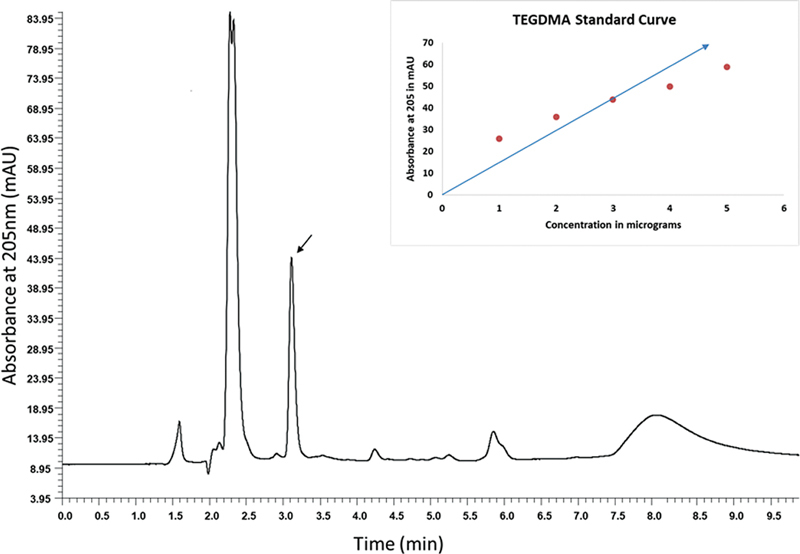
Representative chromatogram obtained after running the standard monomer TEGDMA (3 µg) at 3.1 minutes. The y-axis represents absorbance at 205 nm in mAu and x-axis represents time in minutes. The *arrow* indicates the peak of TEGDMA. Inset: Standard curve of concentration versus absorbance of monomer TEGDMA.

The reference curve of standard TEGDMA was prepared using the monomer TEGDMA (Sigma, Aldrich). The TEGDMA monomer concentration was calculated with the variables obtained from linear regression analysis of the results from the calibration curve. A total of 10 µL of solution with 1 to 5 µg of TEGDMA was injected into the HPLC system for obtaining standard chromatogram.( Fig. 1 ) The calibration curve was obtained with reference to increasing absorbance relative to the TEGDMA concentration.

After preparation, the disks were immersed to 2 mL of respective storage solvents in the Eppendorf tubes. Directly after polymerization, the samples were weighed using the analytical balance and were placed in the Eppendorf tubes in 2 mL of extraction solvent: distilled water, 70% ethanol/water solution, and artificial saliva with betel nut extract. The specimens were then placed in the incubator at a temperature of 37°C. After 1 day, the sample was removed from the incubator (Heraeus B6 incubator 220 V, 50 Hz, 1.4 A, 320 W) and applied to an HPLC analysis. Ten microliters of the sample from the Eppendorf tubes was loaded to the column for evaluation of the eluted monomer TEGDMA. After running the sample of 1 day incubation, the extraction media was refreshed and the disks were left in the extraction media for 7 days. Similarly, after the 7-day analysis, the media was refreshed and the samples were then kept in the incubator at 37°C again. The last analysis was carried out after 30 days, by evaluating the release of monomer TEGDMA in all samples through HPLC.

## Results

**Fig. 2:**
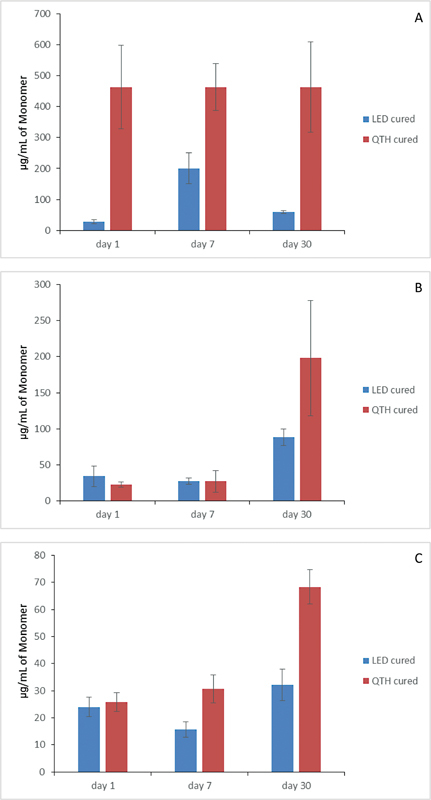
Representative bar diagram of effect of ethanol on ( **A** ) Filtek Z350XT, ( **B** ) Beautifil-Bulk Shofu, and ( **C** ) Filtek Bulk fill flowable 3M ESPE treated with light emitting diode (LED) or quartz tungsten halogen (QTH).

**Fig. 3:**
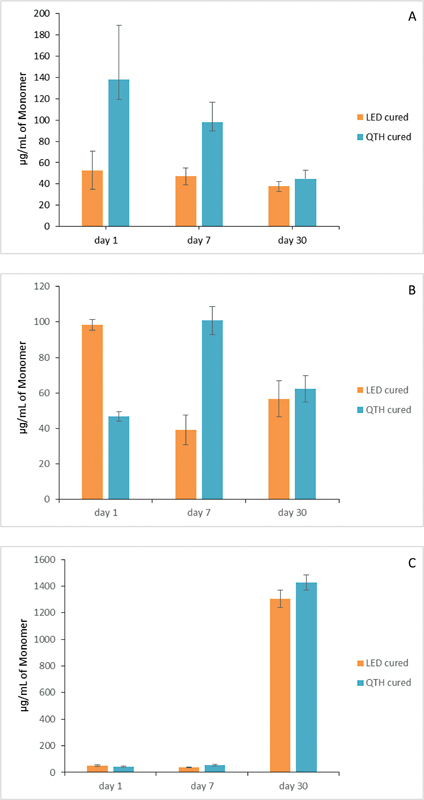
Representative bar diagram of effect of betel quid in artificial saliva on ( **A** ) Filtek Z350XT, ( **B** ) Beautifil-Bulk Shofu, and ( **C** ) Filtek Bulk fill flowable 3M ESPE treated with light emitting diode (LED) or quartz tungsten halogen (QTH ) .

**Fig. 4:**
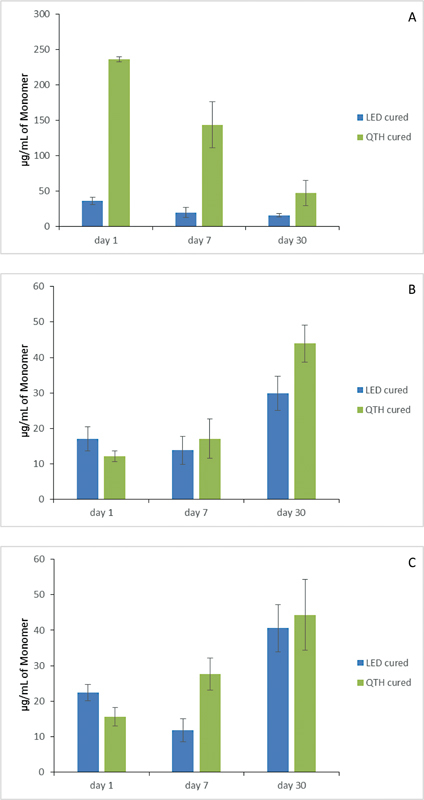
Representative bar diagram of effect of distilled water on ( **A** ) Filtek Z350XT, ( **B** ) Beautifil-Bulk Shofu, and ( **C** ) Filtek Bulk fill flowable 3M ESPE treated with light emitting diode (LED) or quartz tungsten halogen (QTH).

The mean and standard deviation values of the three types of composites are described in Tables 3 4 5 , according to the storage solutions used and the curing lights applied. The elution of the monomer TEGDMA has been shown after immersion in ethanol, artificial saliva, and distilled water in Figs. 2 , 3 , and 4 , respectively, for the three composites at different time intervals.

**Table 3:** Mean value and standard deviation (± SD) of the concentrations (µg/mL) of TEGDMA released from Filtek Z350XT 3M ESPE

	Ethanol			Artificial saliva + betel quid extract			Distilled water		
Time	1 d	7 d	30 d	1 d	7 d	30 d	1 d	7 d	30 d
LED cured	27.6 ± 6.6	200.5 ± 49.6	59.3 ± 4.1	52.7 ± 17.9	47.1 ± 8.1	37.6 ± 4.6	36.4 ± 5.2	20.1 ± 6.8	16.1 ± 2.4
QTH cured	462.6 ± 135	792.5 ± 76	666.2 ± 146.1	138 ± 51	98.2 ± 18.5	44.8 ± 8.3	235.8 ± 3.8	143.5 ± 32.3	47.1 ± 17.8

Abbreviations: LED, light emitting diode; QTH, quartz tungsten halogen; TEGDMA, triethylene glycol dimethacrylate.

**Table 4:** Mean value and standard deviation (± SD) of the concentrations (µg/mL) TEGDMA released of Beautifil-Bulk Shofu

	Ethanol			Artificial saliva+ betel quid extract			Distilled water		
Time	1 d	7 d	30 d	1 d	7 d	30 d	1 d	7 d	30 d
LED cured	34.0 ± 24.4	27.3 ± 4.5	88.2 ± 11.8	98.4 ± 3.1	39.1 ± 8.3	56.64 ± 10.2	17.0 ± 3.4	13.8 ± 3.9	29.9 ± 4.8
QTH cured	22.3 ± 3.6	27.1 ± 14.9	198.12 ± 79.8	46.8 ± 2.7	100.8 ± 7.9	62.21 ± 7.4	12.1 ± 1.5	17.1 ± 5.6	43.89 ± 5.2

Abbreviations: LED, light emitting diode; QTH, quartz tungsten halogen; TEGDMA, triethylene glycol dimethacrylate.

**Table 5:** Mean value and standard deviation (± SD) of the concentrations (µg/mL) TEGDMA released of Filtek Bulk fill flowable 3M ESPE

	Ethanol			Artificial saliva + betel quid extract			Distilled water		
Time	1 d	7 d	30 d	1 d	7 d	30 d	1 d	7 d	30 d
LED cured	24.0 ± 3.5	15.7 ± 2.9	32.12 ± 5.8	49.7 ± 5.4	39.2 ± 2.0	1,306.30 ± 65.0	22.4 ± 2.3	11.8 ± 3.3	40.57 ± 6.6
QTH cured	25.8 ± 3.4	30.7 ± 5.2	68.36 ± 6.3	43.7 ± 4.1	53.5 ± 5.7	1,428.62 ± 57.9	15.6 ± 2.6	27.6 ± 4.5	44.32 ± 10.2

Abbreviations: LED, light emitting diode; QTH, quartz tungsten halogen; TEGDMA, triethylene glycol dimethacrylate.

In the control group, Filtek Z350XT 3M ESPE, the highest elution was observed in the disks cured with the QTH lamp with the highest value observed in ethanol (666.2 ug/ml ± 146.1) after 30 days of immersion. For Beautifil-Bulk Shofu, the highest value measured was of the disks cured with QTH lamp immersed in ethanol after 30 days of storage (198.12 ± 79.8). In case of Filtek Bulk fill flowable 3M ESPE, the release of TEGDMA was highest in artificial saliva after 30 days of storage (1,428 ± 57.9) after curing with the QTH lamp.

Two-way mixed analysis of variance (ANOVA) was conducted to access the difference in concentration of storage material over time with respect to curing light and storage material in the control group. There is a significant effect of time on concentration values ( *p* < 0.001). There are no statistically different values observed with respect to curing lights in the control group. The storage chemicals have a statistically significant effect ( *p* < 0.001), where ethanol shows highest concentrations. In case of Beautifil-Bulk Shofu, the effect of time on concentration is significant ( *p* < 0.001). The effect of curing light and storage material is statistically significant ( *p* < 0.001). In Filtek Bulk fill flowable 3M ESPE, the effect of time on concentration is significant ( *p* < 0.001). The effect of curing light and storage material is also statistically significant ( *p* < 0.001).

When the post hoc test was applied on the data, for a comparison of groups, there was a statistically significant difference ( *p* < 0.01) in the mean concentrations of Filtek Z350XT 3M ESPE and Beautifil-Bulk Shofu. On the other hand, the difference in mean concentrations of Filtek Z350XT 3M ESPE and Filtek Bulk fill flowable 3M ESPE was found to be nonsignificant. While comparing the two experimental groups, there was a significant difference ( *p* < 0.01) in the mean concentrations. This shows that there is considerable difference in the properties of the two experimental groups.

## Discussion

Different studies have been conducted to check the elution of monomers by immersing them into different storage solutions. 6 15 16 17 The results from the current studies concluded that the elution of the monomer TEGDMA depends upon the storage media used and irradiance lamp used for curing of the resin-based composites. Furthermore, this study limits itself to the effect of artificial saliva, distilled water, and ethanol, and did not use an acidic environment that is created by the cariogenic bacteria on the tooth surface.

HPLC was used to evaluate the release of the monomer TEGDMA from one conventional and two bulk-fill resin-based composites. The disks were immersed for 1, 7, and 30 days and TEGDMA was found in all the solvents when evaluated using HPLC. This is in agreement with other studies, which found TEGDMA to be the major monomer eluted from different composites. 2 6 18 19

Tsitrou et al 15 discovered that low-molecular-weight monomers could be extracted in considerably higher quantities than high-molecular-weight monomers. Low-molecular-weight monomers such as TEGDMA have higher mobility and will be eluted faster than large molecules like Bis-GMA and UDMA. 18

Durner et al reported that the elution of TEGDMA was high due to its hydrophilic nature and its low molecular weight, which tends to increase its mobility and thus make the elution easier. 20 In the present study, TEGDMA was found to be eluted after 24 hours and 7 days. But the long-term immersion also showed considerable amounts of eluted TEGDMA. 12

The DC for dental composites is approximately between 35 and 77%. Asmussen and Peutzfeldt 21 reported that a linear polymer structure with a fewer cross-links is significantly more prone to softening in ethanol. Thus, incomplete polymerization may increase residual monomer. 1 Maximum elution, that is, 85 to 100% of monomers, was reported to occur within 24 hours by Ferracane 2 and within 7 days by Örtengren et al. 22 23 Hence, the elution of monomers after polymerization was tested at the end of 24 hours and 7 days in the present study. For long-term release of the monomer TEGDMA, Alshali et al 6 stated that the elution of monomers Bis-GMA and UDMA usually gets completed after 3 months, whereas monomer TEGDMA elution nearly gets completed after 1 month. An important factor to be considered in the residual monomer is the molecular weight and the size of the monomer. TEGDMA, being a smaller molecule, tends to release more and quickly as compared to large monomers including Bis-GMA. 23

In the present study, the elution of TEGDMA has been evaluated after a 1-month immersion and all the three types of composites showed considerable amounts of monomer elusion.

Both LED and QTH lights are used for curing the resin-based composites. The samples cured with the QTH lamp have shown increased elution of TEGDMA when compared with the samples cured with LED light. Clinically, the most common strategy to maximize DC and minimize monomer elution is to provide sufficient energy to the system by increasing curing time. Several works focusing on commercially available composites have emphasized the need to apply at least 20 seconds, but more likely 40 seconds, of irradiation to minimize the amount of eluted substances, even with the use of high-irradiance LED lights. 3 24 As stated by Polydorou et al, 25 40 seconds of polymerization time with a halogen lamp is indicated by the manufacturers to achieve adequate polymerization of 2-mm incremental thickness of composite. But the findings of this study revealed that curing time has no significant effect on TEGDMA elution. The polymerization time was kept to 40 seconds in our study and monomer elution has been observed in all storage solutions. According to Polydorou et al, 26 for the elution of TEGDMA, no difference was found between the 20-, 40-, and 80-second polymerization for all three different storage periods tested. Therefore, it can be concluded that the 40-second polymerization that is usually used as a polymerization time, which is thought to have satisfying results in the mechanical properties of the composite resins, compared to the 20-second polymerization time, does not seem to be more effective on the release of monomers.

We can distinguish two groups of solvents: water or aqueous mixtures, such as cell culture media artificial saliva and human saliva, and different organic extraction media, such as ethanol, methanol, acetone, acetonitrile, tetrahydrofuran, and chloroform. 13 The type of extraction medium used impacts both the concentration of the eluted monomer and the elution time. In general, the degree of monomer elution is proportional to the hydrophobicity and swelling capacity of the organic solvent used. In our study, we have used 70% ethanol/water solution, artificial saliva with betel nut extract, and distilled water. However, according to the International Organization for Standardization (ISO) specification, distilled water is an extraction medium for resin-based filling materials, which simulates a humid, intraoral environment containing both saliva and water. 27 The U.S. Food and Drug Administration (FDA) has graded a 75% ethanol–water solution as clinical oral stimulating liquid and it has been used in several studies. 28 In our study, we incorporated a 70% ethanol–water solution as one of our storage solutions and the elution of TEGDMA was high in ethanol as compared to other solutions. It can further be justified by the fact that ethanol has the ability to penetrate within the unreacted monomer and widens the gap within the polymer chains, allowing the soluble compounds to elute. It can mimic and accelerate the typical degradation as expected clinically from the food and saliva through continuous exposure. 29 The amount of TEGDMA monomer released according to different authors was 0.005 to 2.424 µg/mL. 12 30 31

Several studies have been conducted to check the cytotoxic and genotoxic effects of these leachable materials from the dental biomaterials. These effects then cause adverse biological reactions including local and systemic cytotoxicity, and pulpal and allergic reactions. 32 Studies reporting the leaching of uncured monomers from dental adhesive system through dentin into the dentinal fluid are scarce. Dentinal fluid is an important component of the pulp–dentin complex, which is a communication between pulp and other areas of dentin. In healthy conditions, the composition of dentinal fluid is controlled by the odontoblasts. However, after stresses such as caries or trauma, the fluid composition is closer to plasma. 32

Inflammation then leads to increased capillary permeability and localized vasodilatation allowing leakage of plasma proteins from the blood flow to dentinal fluid. This transudates from the exposed dentin and contains proteinlike fibrinogen, immunoglobulin G (IgG), and albumin. 33 Albumin is a multifunctional, low-molecular-weight taxi protein that has the ability to bind and transport almost any small molecule. According to Mahdhaoui et al, it can be stated that albumin can transport unbound monomers released from dental adhesives through the dentin barrier. 32 It is believed that basic monomers such as Bis-GMA and TEGDMA comonomer have got a toxic potential. TEGDMA, apart from the modified basic monomers UDMA and bis-EMA, is the main comonomer of the SDR composite resin. TEGDMA is one of the most frequently used diluents in the composite materials. Its low molecular mass and the presence of ethylene oxide groups make this monomer reactive, mobile, and relatively easy to elute from the composite material matrix. 13 However, the incorporation of nanoparticles and combination of Bis-GMA have been reported to enhance mechanical as well as biological properties. 34 35 Other materials like halloysite nanotubes have also been shown to provide similar enhancement and antibacterial properties. 36 37

The unreacted TEGDMA is a toxic substance exhibiting cytotoxic, genotoxic, mutagenic, and allergenic effects. Directly capping the pulp with the use of composite resins does not lead to dentine bridge formation and may be one of the reasons for the development of inflammatory reactions in dental pulp cells, their apoptosis, as well as dental pulp inflammation and necrosis. 38 39 40 The effective dose (ED50) for TEGDMA, assessed in human dental pulp fibroblasts cultures, is about 0.08 mg/mL. Therefore, similar or higher monomer concentrations, without sufficient protection of the bottom of cavity, may lead to dental pulp injuries. Unreacted TEGDMA monomer may also be a substrate for microorganisms colonizing the marginal gap. It promotes the proliferation of cariogenic microorganisms. 13

There are no unequivocal literature data on the time period necessary for total elution of unreacted TEGDMA monomer from composite material. According to some studies, the elution process ends after 1 to 7 days, while other authors state that it is longer, for example, 30 days. 25 41 42 In the present study, elution of the monomer TEGDMA was the highest after immersion for 24 hours in a 70% ethanol/water solution, artificial saliva, betel nut extract, and distilled water. It has been reported previously that the release of TEGDMA from the composite may stimulate the growth of cariogenic bacteria and could lead toward secondary caries, 43 a reason not to include the acidic solution for monitoring the elution pattern of the monomer TEGDMA in this study as mentioned earlier. Ferracane 2 report that 50% of monomers are eluted from material during the first 3 hours after polymerization and 85 to 100% of monomers are eluted within 24 hours. 44 More recent studies using HPLC have shown that monomer elution continued beyond 24 hours for resin-based composites. 26 45 However, despite further potential monomer elution, the majority of soluble substances are extracted from the material within hours.

## Conclusion

Within the limitations of the study, it has been concluded that Filtek Bulk fill flowable 3M ESPE showed a lower release of the TEGDMA monomer into the storage media. The material cured by the QTH lamp results in increased release of monomer. The distilled water has been the most stable storage media in which the cured samples were immersed to check the monomer release.

## Recommendations

The use of LED light to cure composite when used in the clinical setup should be preferred over the QTH lamps. Filtek Bulk fill flowable 3M ESPE is advisable to be used within the clinical setup as it releases less monomer with the passage of time. There had been tremendous release of the TEGDMA monomer in artificial saliva with betel quid when Filtek Bulk fill flowable 3M ESPE was immersed for 1 month, so more studies can be conducted on this in future.

## Limitations

- Only one type of monomer, TEGDMA, was used to study the elution, due to the lack of availability of other monomer standards and toxicity, for instance Bis-GMA.
- The study did not perform any techniques to check the DC of a monomer into a polymer, for example, the Raman spectroscopy.
